# Experiment and Theory Clarify: Sc^+^ Receives One Oxygen Atom from SO_2_ to Form ScO^+^, which Proves to be a Catalyst for the Hidden Oxygen‐Exchange with SO_2_

**DOI:** 10.1002/cphc.202100773

**Published:** 2022-01-20

**Authors:** Jose M. Mercero, Elixabete Rezabal, Jesus M. Ugalde, Thomas Weiske, Jilai Li

**Affiliations:** ^1^ Kimika Fakultatea, Euskal Herriko Unibertsitatea (UPV/EHU), and Donostia International Physics Center (DIPC) P. K. 1072 Donostia 20080 Euskadi Spain; ^2^ Institut für Chemie Technische Universität Berlin Straße des 17. Juni 115 10623 Berlin Germany; ^3^ Institute of Theoretical Chemistry Jilin University 130023 Changchun China

**Keywords:** catalysis, gas-phase reactions, Mars-van Krevelen mechanism, oxygen-atom exchange, quantum chemical calculations

## Abstract

Using Fourier‐transform ion cyclotron resonance mass spectrometry, it was experimentally determined that Sc^+^ in the highly diluted gas phase reacts with SO_2_ to form ScO^+^ and SO. By ^18^O labeling, ScO^+^ was shown to play the role of a catalyst when further reacting with SO_2_ in a Mars‐van Krevelen‐like (MvK) oxygen exchange process, where a solid catalyst actively reacts with the substrate but emerges apparently unchanged at the end of the cycle. High‐level quantum chemical calculations confirmed that the multi‐step process to form ScO^+^ and SO is exoergic and that all intermediates and transition states in between are located energetically below the entrance level. The reaction starts from the triplet surface; although three spin‐crossing points with minimal energy have been identified by computational means, there is no evidence that a two‐state scenario is involved in the course of the reaction, by which the reactants could switch from the triplet to the singlet surface and back. Pivotal to the oxygen exchange reaction of ScO^+^ with SO_2_ is the occurrence of a highly symmetric four‐membered cyclic intermediate by which two oxygen atoms become equivalent.

## Graphical abstract

**Sc^+^ is oxidized by SO_2_ to ScO** ^+^, which catalyzes the hidden oxygen exchange reaction with SO_2_ in a Mars‐van Krevelen‐like reaction.

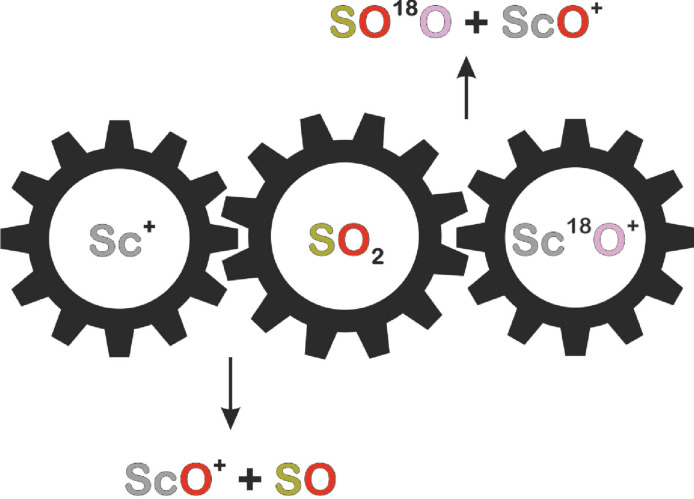

## Introduction

Sulfur dioxide is a colorless, mucous membrane‐irritating, pungent‐smelling and sour‐tasting toxic gas. The largest amount of the sulfur dioxide, caused by human activities, released into the atmosphere comes mainly from the combustion of sulfur‐containing fossil fuels such as coal or petroleum products and it contributes significantly to global air pollution. However, it is worth noting that sulfur dioxide is also a natural byproduct of volcanic activity.

When its concentration in the air exceeds the safety threshold, sulfur dioxide harms humans, animals and plants.[^1^, ^2^] Atmospheric sulfur dioxide, along with other SO_*x*_ oxides, are also culpable for the formation of smog as well as acid rain,^[3]^ by which forests and lakes can be severely damaged.^[4]^ Fortunately, in Europe and North America, the amount of sulfur dioxide of man‐made origin released into the atmosphere has been reduced by 70–80 % over the past 30 years,^[5]^ and consequently, the removal of atmospheric sulfur dioxide has recently gained much interest.^[6]^ In this vein, the so‐called direct sulfur recovery process (DSRP)^[7]^ constitutes a long‐sought goal.[^8^, ^9^, ^10^] DSRP is aimed at chemically reducing sulfur dioxide to elemental sulfur, so that it becomes a valuable chemical feedstock.

Earlier studies on the catalytic oxygen‐sequestration from SO_2_ by transition metal complexes suggest that it can be a suitable route towards the chemical reduction of SO_2_.^[11]^ In this vein, Armentrout et al., using guided ion beam mass spectrometry,^[12]^ have reported that the activation of the S−O bond of sulfur dioxide by the cations of the heavy transition metals rhenium,^[13]^ osmium,^[14]^ and iridium^[15]^ can only be achieved under endothermic conditions. Naturally, for most of the considerations in this undertaking, the S−O bond activation represents the key issue.^[16]^ Consequently, a better understanding of the SO_2_ activation and S−O bond cleavage by transition metals is vital in order to make progress in this field.

Gas phase mechanistic studies are ideally suited for this purpose, for experiments carried out with mass‐selected species at their electronic ground states under single‐collision conditions, avoid all interferences due either to solvent or surface environments, and provide clean, chemically relevant information about the specific role of the selected transition metal in its interaction with the target substrate. The oxidation number, charge and spin states, etc. of the catalytically active metal can be interrogated without being affected by unknown effects due to the presence of a poorly characterized environment. It has repeatedly been shown that gas‐phase experiments of this kind are the most suitable approach to investigate the thermodynamics and kinetics of chemical reactions at a strictly molecular level.^[17]^ When this type of experimental information is then complemented by quantum electronic structure studies at a sufficiently high level, a valuable conceptual framework emerges that allows specific mechanistic questions about the role of the active catalytic site of the reaction to be addressed.

In the present work, such an approach is pursued to study the reactions of scandium cations with sulfur dioxide.

### Inventory: What is Already Known About the Main Actors Sc^+^ and ScO^+^

Scandium is the simplest transition metal element with only one 3d electron. Scandium cation, then, could be seen as the simplest transition metal cation with no 3d electrons. However, Sc^+^ does have one 3d electron for its ground‐state electronic configuration is 3p^6^3d^1^4s^1^. The 3p^6^3d^0^4s^2^ configuration, which has no 3d electrons, lies 140 kJ/mol (11.736.26 cm^−1^) higher in energy,^[18]^ and therefore will be virtually absent from the composition of ions reacting at room temperature. This leaves the reacting Sc^+^ ions with two unpaired electrons, one in the 3d and one in the 4s orbital, which allows for both, the ferromagnetic spin coupling, which yields the electronic ground state ^3^D, and the antiferromagnetic spin coupling, which yields the ^1^D as the first electronic excited state (vide infra), separated by 30 kJ/mol (2,540.95 cm^−1^).^[18]^

The reactivity of Sc^+^ in the gas phase has been extensively studied in the past and has produced a massive amount of chemical literature with an emphasis on the selective activation of halides, alkanes and alkenes[^19^, ^20^, ^21^, ^22^, ^23^, ^24^] and the dehydrogenation of water.[^25^, ^26^, ^27^, ^28^] Activation of SO_2_ by Sc^+^ has not been studied yet. Notice, however, that the experimentally determined bond dissociation energies (BDE) at T=298 K of ScO^+^ and SO_2_ amount to 692±5 and 551±1 kJ/mol, respectively,[^29^, ^30^] Therefore, the oxygen abstraction reaction Sc^+^+SO${}_{2}\to$ [ScO]^+^+SO is exothermic by 141±6 kJ/mol and could well proceed from a purely thermodynamic point of view. However, there still remains the possibility that this reaction is prevented by a sufficiently high energy barrier for kinetic reasons.

Scandium oxide cation, [ScO]^+^, has been generated experimentally in the past by guided ion beam experiments,^[29]^ and its reaction with deuterium^[31]^ and with methane,[^32^, ^33^] have been reported. Scandium oxide cation, [ScO]^+^, has also been the subject of several quantum chemical calculations.[^34^, ^35^, ^36^] Indeed, based on these calculations, an interesting catalytic cycle was proposed in which CO is oxidized to CO_2_ by NO_2_, mediated by Sc^+^ which facilitates the formation of the [ScO]^+^ intermediate.^[37]^ As far as we are aware of, there are no reports on investigations about reactions of Sc^+^ and ScO^+^ with SO_2_, in the open chemical literature.

### Experimental Results on the Reaction of Sc^+^ with SO_2_

Scandium cations, (Sc^+^), have been formed by the supersonic expansion of helium into a scandium plasma generated by laser ablation/ionization of a rotating scandium disk made of natural scandium, which consists by 100 % of the isotope ^45^Sc, using a Nd:YAG laser, operating at 532 nm inside the external cluster source of a Fourier‐transform ion cyclotron resonance (FT‐ICR) mass spectrometer as described previously (for details, see the Supporting Information).[^38^, ^39^, ^40^] A fraction of the ion population was then guided by a static ion optical system into the ICR cell. Next, in a sequence of pulses, argon was admitted to the ICR cell such that the ions collide on average about 1×10^5^ times with argon. This procedure ensures thermalization of hot ions and quenching of excited electronic states. After mass‐selection of the thermalized Sc^+^ ions, they were reacted with SO_2_ at a constant pressure low enough to ensure single collision conditions. The elementary compositions of the charged species have been confirmed by high‐resolution mass spectrometry.

**Figure 1:**
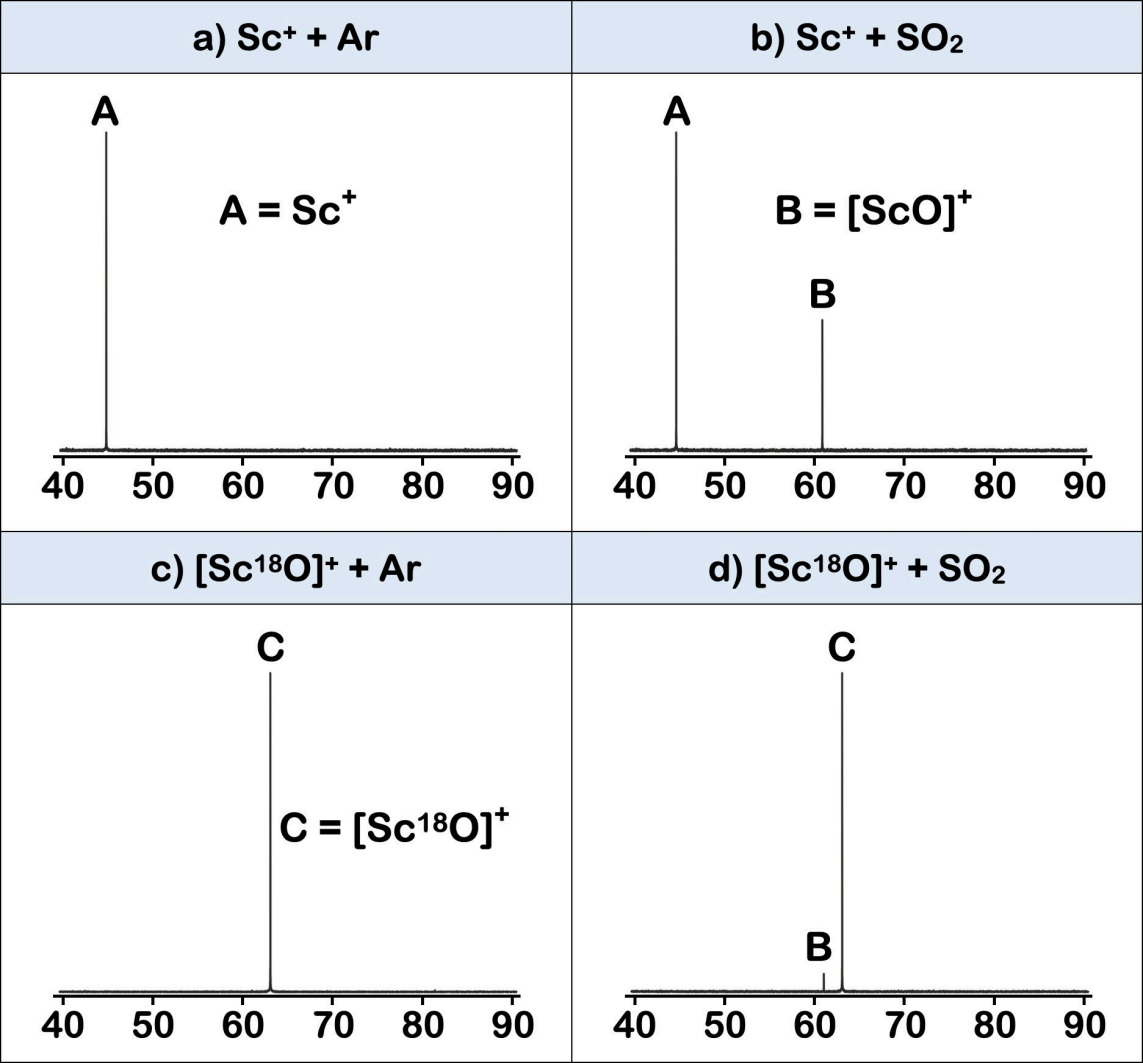
Representative mass spectra for the reactions of Sc^+^ with Ar (a) and SO_2_ (b) and of [Sc^18^O]^+^ with Ar (c) and SO_2_ (d) at ambient temperatures and 1.5×10^−9^ mbar after a reaction time of 5 s. All *x*‐axes are scaled in m/z , and the *y*‐axes are normalized relative ion abundances.

The result of the reaction of Sc^+^ with SO_2_ at a partial pressure of 1.5×10^−9^ mbar and a reaction time of 5 s inside the ion trap of the FT‐ICR machine is shown in Figure 1b. In addition to the signal of the starting material Sc^+^ (signal A), a signal B appears, which has been assigned the formula [ScO]^+^ by exact mass measurements. Clearly, oxygen atom transfer (OAT) from SO_2_ to the scandium ion takes place as formulated in Eq. 1.

(1) $$\mathrm{Sc}{}^{+}+\mathrm{SO}{}_{2}\;\to \;\left[\mathrm{ScO}\right]{}^{+}+\mathrm{SO}$$

In a next step, it was examined whether the product ion [ScO]^+^ is able to further react with SO_2_ via an oxygen‐exchange process according to Eq. 2.

(2) $$\left[\mathrm{Sc}{}^{18}O\right]{}^{+}+\mathrm{SO}{}_{2}\;\to \;\left[\mathrm{ScO}\right]{}^{+}+\mathrm{SO}{}^{18}O$$

Since reactant and product ions would not otherwise differ in mass, oxygen labeling is required in this case. The [Sc^18^O]^+^ used here has been generated inside the external cluster ion source by adding trace amounts of ^18^O_2_ to the expanding helium buffer gas. Figures 1c and d display the outcome of this labeling experiment. Indeed, the appearance of a signal corresponding to unlabeled [ScO]^+^ (signal B in Figure 1d) starting from [Sc^18^O]^+^ and SO_2_ clearly proves that the oxygen exchange reaction between scandium oxide and sulfur dioxide, as formulated according to Eq. (2), occurs.

This finding constitutes one more example of the Mars‐van Krevelen (MvK) mechanism,[^41^, ^42^, ^43^, ^44^, ^45^, ^46^] a term that belongs to the basic concepts of heterogeneous catalysis,^[46]^ although the experiments performed here lie within the scope of homogeneous catalysis. The reaction between Sc^+^ and SO_2_ is found to belong to the very rare cases in which the catalytically active species, namely the [ScO]^+^ oxide cation, can be identified.^[46]^ Moreover, [ScO]^+^ is, in the best sense, a prime example of a catalyst that emerges seemingly unchanged after the reaction has been completed.

The rate constants *k*_1_(Sc^+^/SO_2_) and *k*_2_([Sc^18^O]^+^/SO_2_) for the reactions of Eqs. (1) and (2) were measured, and they amount to 6.9×10^−10^ (efficiency *ϕ*=38 %) and 2.2×10^−10^ cm^3^ molecule^−1^ s^−1^ (*ϕ*=12 %), respectively. Owing to an uncertainty in the determination of the absolute pressure of SO_2_, an error of ±30 % is associated with the rate measurements.^[39]^ By experiment so far it has been clarified what processes occur in the course of the reactions of Sc^+^ and ScO^+^ with SO_2_ at ambient temperature in the highly diluted gas phase. What remains is to shed light into the details of the mechanisms involved. This is best accomplished using high level quantum chemical calculations.

### Remarks on the Applied Quantum Chemical Methods

Exploratory calculations for the reaction given by Eq. (1) at the CCSD(T)/TZVP+ level of theory yielded T1‐diagnostic values that far exceed the recommended value of 0.05 for reliable single‐reference calculations of the electronic structure of species containing first‐row transition metals.[^47^, ^48^, ^49^]

Thus, given the large multiconfigurational nature of the reactants, products, and intermediates involved, in order to obtain trustworthy results, it proved necessary to use multi configurational perturbation theory on a carefully energy optimized multi configurational self‐consistent field wave function with a triple‐*ζ* quality basis set (see Section 2 of the Supporting Information for a detailed account of the theoretical methods employed herein) to investigate the mechanism of the reaction shown in Eq. (1). Hence, we opted for the multiconfigurational quasi‐degenerate perturbational calculations on the multiconfigurational self‐consistent field wave function expanded on an active space with 14 valence electrons in 15 active molecular orbitals, with the TZVP+ basis set,^[27]^ which will be denoted as MCQDPT/TZVP+//MCSCF(14,15)/TZVP+ hereafter.

One means of assessing the reliability of theoretical results rests on comparing calculated excitation energies of the various states of Sc^+^ with experimentally measured data. The 3p^6^3d^1^4s^1^ configuration is that with the lowest energy of Sc^+^, which yields the ^3^D and ^1^D terms. The former is further split by the spin‐orbit coupling into the J=1, 2, and 3 levels, while the latter yields the J=2 level. The excitation energies of all these levels have been measured with high precision. Our theoretical procedure reproduces all of them with remarkable accuracy (See Table S3 of the Supporting Information for details). Note that the calculated value for the ^3^D (J=1) to ^1^D (J=2) excitation given here differs by less than 2 kJ/mol from the experimentally obtained one, while CCSD(T) calculations^[27]^ miss it by 16 kJ/mol, which indicates that the results from CCSD(T) may not be sufficiently reliable. Furthermore, we have also estimated the dissociation energies of ScO^+^ and SO_2_ to be respectively 673.4 kJ/mol and 552.4, which compare satisfactorily with their corresponding experimental marks, namely 692±5 kJ/mol and 551±1 kJ/mol (see Section 2).

### Mechanism of the Oxygen‐Abstraction from SO_2_ by Sc^+^

**Figure 2:**
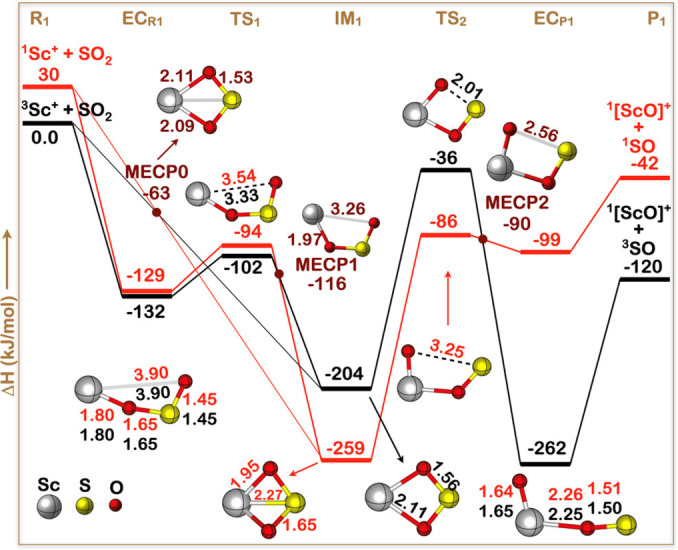
Schematic potential energy surface, in kJ/mol, calculated at the MCQDPT/MCSCF(14,15)/TZVP+ level of theory for the reaction Sc^+^+SO_2_→[ScO]^+^+SO. The geometrical features of the singlet and triplet states of all the intermediates, except **TS_2_**, are indistinguishable on this scale. Refer to Tables S1 and S4 for further details. The color codings of selected bond distances, in Å, shown are: red for singlet and black for triplet.

Figure 2 shows a combined plot of the energy‐minimized profiles of the singlet and triplet potential energy surfaces (PES's). The first general observation here is that the reaction is predicted to be exoergic regardless of the spin state, be it singlet, or be it triplet. And second, again independent of the spin state, there is no kinetic barrier in between that could prevent the reaction at ambient temperature.

The ground states of the reactants correspond to the ground triplet spin‐state of Sc^+^ (^3^D, 3p^6^3d^1^4s^1^) and the ground singlet spin‐state of SO_2_ (^1^A_1_). The first excited electronic state of Sc^+^ is the singlet spin state ^1^D (3p^6^3d^1^4s^1^), which lies 30 kJ/mol (2,540.95 cm^−1^) higher than its ground triplet spin state (see Table S3). The corresponding first excited electronic state of SO_2_ is the ^3^B_1_ triplet spin state which is 310 kJ/mol (25,929.07 cm^−1^) higher in energy[^50^, ^51^] than its ground singlet spin state. In view of these excitation energies, and since Sc^+^ ions were thoroughly thermalized before reacting with SO_2_, as already mentioned in the experimental section, it is very unlikely that the excited singlet spin state of Sc^+^ to be populated in an amount as to have any effect on the outcome of the experimental results.

On the products side, their ground states consist of the singlet spin state ${}^{1}\Sigma^{+}$ in case of ScO^+^, and the triplet state (${}^{3}\Sigma^{-}$ ) in the case of SO. The first excited state of ScO^+^ is the ${}^{3}\Delta$ triplet, which is well above the ground state by 389 kJ/mol (32,531.5 cm^−1^).^[52]^ The lowest excited states a${}^{1}\Delta$ and b${}^{1}\Sigma^{+}$ of SO are energetically higher by 76 kJ/mol (6,350.0 cm^−1^) and 126 kJ/mol (10,510.0 cm^−1^), respectively.^[53]^

Under our experimental conditions, vide supra, the reactants are prepared on the triplet PES. Thus, in accordance with the reaction mechanism sketched in Figure 2, the reaction will commence with the formation of the encounter complex **EC**_R1_, which subsequently rearranges via transition state **TS**_1_ to the symmetric four‐membered ring‐intermediate **IM**_1_ comprising butterfly structure. Theoretically, the triplet and singlet of the reactants could directly merge into singlet and triplet of the intermediate **IM**_1_ by bypassing **EC**_R1_ via the minimum energy spin‐crossing point **MECP0** (see Table S4 in the Supporting Information), thereby swapping their spin‐multiplicities, but since the coupling constant of the spin‐orbit coupling effect^[54]^ is very small, only 0.94 cm^−1^ at **MECP0** (see Table S4), a transition between the PES's of the triplet and singlet spin states can be ruled out practically.

After surmounting the relatively high barrier of **TS**_2_, the triplet spin‐state of the cyclic intermediate **IM**_1_ opens to the exit‐channel complex **EC**_P1_, which finally dissociates into the ground states of the products ScO^+^ and SO.

Remarkably, the C_*s*_ symmetry is conserved during the course of the reaction, although the reaction involves four atoms. All the intermediate structures belong to the in‐plane A′ irreducible representation. We have searched for the A′′ symmetry states of all the intermediate stable structures. However, our MCSCF(14,15)/TZVP+ calculations reveal that they should lie much higher in energy, for their relative energies with respect to their corresponding A′ states range from 183 kJ/mol, for EC_R1_, to 409 kJ/mol for the triplet state spin‐state of the EC_P1_ intermediate. Recall, nonetheless, that MCSCF wavefunctions simply constitute a reference wave function for subsequent calculations of molecular properties.^[55]^ Thus, these energies should not be taken as accurate relative energies. But, for values this large, one can safely state that the A′′ states are excited states. Finally, we would like to emphasize that the lowest energy structure along the reaction path outlined in Figure 2, namely, the triplet spin‐state of intermediate **EC**_P1_, features the highly unstable SO sulfur oxide stabilized by a transition metal. Indeed, the SO, along with S_2_O and S_2_O_2_, are known to be stable only in complex‐bound form.^[56]^ Our calculations suggest that ScO^+^ may be a suitable stabilizer in order to pursue more detailed studies^[57]^ on sulfur monoxide, which is highly reactive and often classified as a “laboratory curiosity”.^[56]^

According to the calculations, the formation of the final products, i. e., singlet ScO^+^ (${}^{1}\Sigma^{+}$ ) and triplet SO (${}^{3}\Sigma^{-}$ ), is exoergic by 120 kJ/mol, in line with our experimental estimate of 141±6 kJ/mol (see Section 2). In contrast, if singlet SO (${}^{1}\Delta$ ) is split off as a neutral particle instead of the triplet under otherwise unchanged conditions, the process would turn out to be exoergic by only ca. 42 kJ/mol. Both energy values refer to the reactants Sc^+^ (^3^D, 3p^6^3d^1^4s^1^) and SO_2_ (^1^A_1_) (see Table S2 of the Supporting Information).

Although two additional minimum energy spin‐crossing points have been identified, represented by the structures **MECP1** and **MECP2** in Figure 2, where triplet and singlet spin PES intersect, a two‐state‐reactivity scenario[^58^, ^59^] can most likely be ruled out. Namely, since intersystem crossing is a spin “forbidden process” – but allowed by the relativistic spin‐orbit coupling – one should expect low efficiency for the oxygen‐abstraction reaction if the spin‐crossing occurs in a large extent, for the overall reaction in such a case would be only marginally exoergic 42 kJ/mol, as indicated above. This, which in principle could be possible in view of the large spin‐orbit coupling constant of 234.07 cm^−1^ calculated for **MECP2** (see Table S4 of the Supporting Information), is contradicted by the relatively high reaction efficiency observed experimentally, which is very suggestive of a large exoergicity for the overall reaction. Thus, our calculations for the oxygen‐abstraction reaction of Eq. (1) predict the production of ScO^+^ (${}^{1}\Sigma^{+}$ ) and SO (${}^{3}\Sigma^{-}$ ). Further confirmation of this prediction could be made by comparing the measured vibrational frequency of the resulting ScO^+^ cation with its experimentally determined value of 976.3 cm^−1^.^[60]^ Note that the formation of ScO^+^ (${}^{3}\Delta$ ) can be excluded, as this would result in an endoergic reaction by about 269 kJ/mol.

### Mechanism of the Oxygen‐Exchange Reaction Between ScO^+^ and a Secondary SO_2_

**Figure 3:**
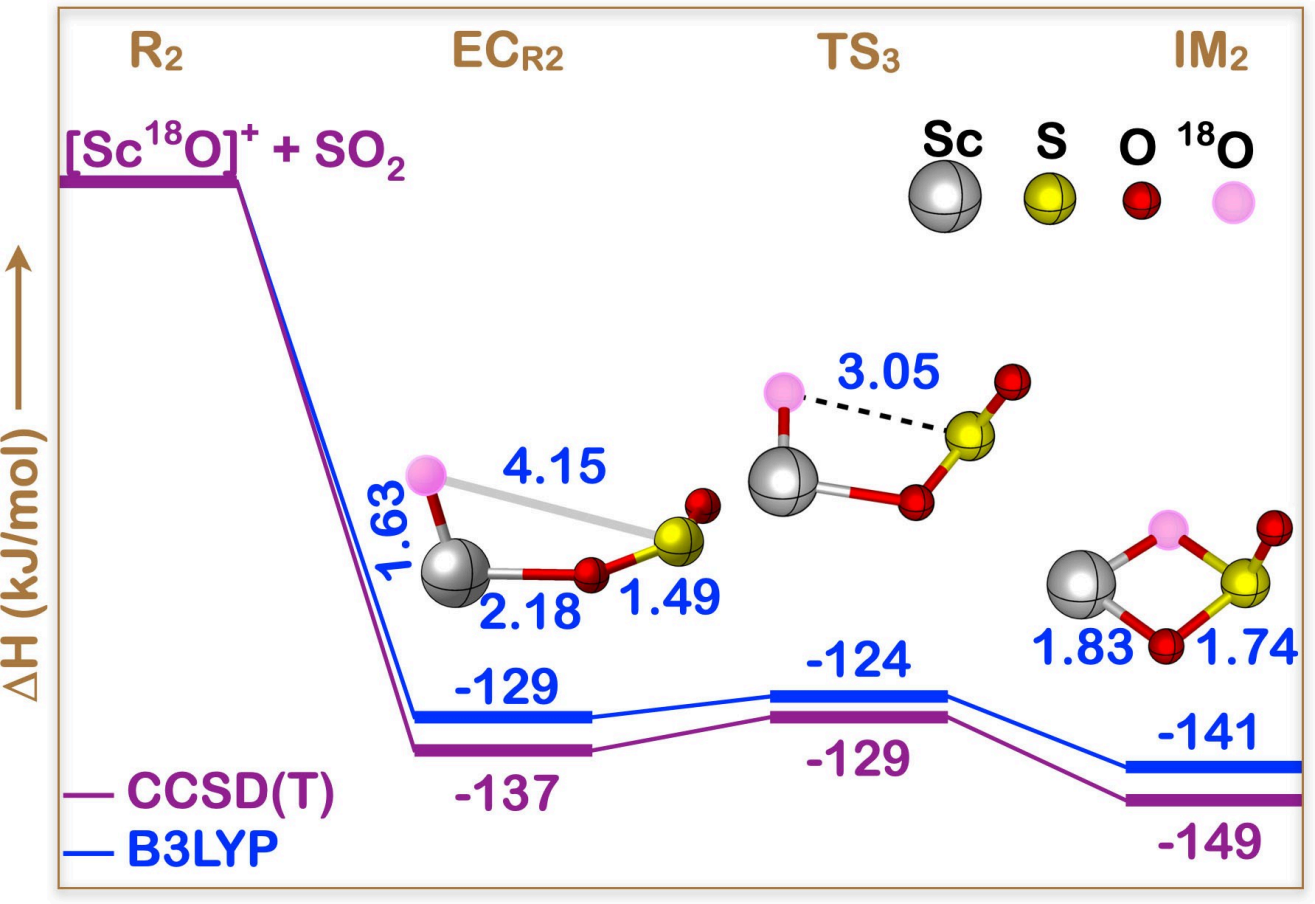
Schematic CCSD(T)/TZVP+//B3LYP/TZVP+ and B3LYP/TZVP+//B3LYP/TZVP+ potential energy surfaces, in kJ/mol, for the reaction [Sc^18^O]^+^+SO_2_→[ScO]^+^+SO^18^O. The selected B3LYP/TZVP+ optimized bond distances shown are in Å. The Sc−^18^O−S−O dihedral angles are, **EC**_R2_: −13.7°, **TS**_3_: 3.1°, and **IM**_2_: 6.3°. Full geometry details are given in Table S6.

According to the experimental results, and as formulated in Eq. (2), ScO^+^ (${}^{1}\Sigma^{+}$ ) can undergo a “hidden” oxygen exchange by reacting with SO_2_. The details of the mechanism behind of such a MvK like process have been investigated theoretically, and will be discussed here. First of all, it is pointed out here that for this case the CCSD(T) method is appropriate to provide a reliable description of the entire reaction pathway, as suggested by the sufficiently low scores (smaller than the recommended 0.05) of the T1 diagnostic values of all reactants, intermediates and products (see Table S5 in the Supporting Information) of the reaction mechanism. Thus, all structures displayed in Figure 3 have been optimized at the B3LYP/TZVP+ level of theory, and their energies refined by CCSD(T)/TZVP+ calculations.

The pivotal point of the oxygen‐exchange process is comprised by the cyclic and C_*s*_‐symmetric intermediate **IM**_2_, which halfway connects the reactants [Sc^18^O]^+^ and SO_2_ to the products [ScO]^+^ and ^18^OSO through the transition state **TS**_3_ and the encounter complex **EC**_R2_ as displayed in Figure 3. Due to the C_*s*_ symmetry both coplanar oxygen atoms of the four‐membered ring of **IM**_2_ become equivalent and on ring reopening via **TS**_3_ and reformation of the encounter complex **EC**_R2_ either the labeled or the unlabeled oxygen atom will remain at the metal center. **EC**_R2_ finally falls apart into neutral sulfur dioxide and the charge bearing scandium oxide cation, which is now ready for another catalytic cycle.

## Conclusions

We have generated laser ablated thoroughly thermalized Sc^+^ cations and let them react with SO_2_. Our experiment shows that the above mentioned oxygen‐abstraction reaction yields cationic scandium oxide, ScO^+^, and neutral sulfur oxide, SO. Subsequent high‐level quantum electronic structure calculations have revealed that the products of the reaction are ScO^+^ (${}^{1}\Sigma^{+}$ ) and SO (${}^{3}\Sigma^{-}$ ), with a calculated exoergicity of 120 kJ/mol relative to the ground state of the reactants, i. e., Sc^+^ (^3^D, 3p^6^3d^1^4s^1^) and SO_2_ (^1^A_1_). Additionally, the calculations have also revealed that the mechanism of such reaction resembles a two‐state reactivity scenario involving two spin‐crossings, which have been deemed unlikely to occur, in spite of the large spin‐orbit coupling constant predicted for the second spin‐crossing structure.

The scandium monoxide cation [ScO]^+^ is a poor oxygen acceptor since the BDE of ScO${}^{+}-$ O is only 166 kJ/mol,^[61]^ and consequently cannot abstract an oxygen atom from SO_2_. However, it can act as a catalyst for the oxygen‐exchange reaction, [Sc^18^O]^+^+SO_2_→[ScO]^+^+SO^18^O, Eq. (2). This, which constitutes the most interesting finding of the present work, has been demonstrated experimentally, and the associated mechanism determined by high‐level quantum electronic structure calculations. The resulting MvK‐like process proceeds through a C_*s*_ symmetric four‐membered cyclic intermediate which renders the exchanging oxygen atoms equivalent, as required for MvK‐like processes.^[46]^

In some catalytic reactions, a pre‐catalyst needs to undergo a transformation to form the active species, before the catalyst can take effect.^[62]^ The Sc^+^/SO_2_ system represents such an example where the actual catalytically active species [ScO]^+^ must be generated first before the catalytic oxygen‐exchange commences. We also expect the present study will provide some useful clues for the deactivation, instability, as well as poor sintering resistance of catalysts.

## Conflict of interest

The authors declare no conflict of interest.

1

## Supporting information

As a service to our authors and readers, this journal provides supporting information supplied by the authors. Such materials are peer reviewed and may be re‐organized for online delivery, but are not copy‐edited or typeset. Technical support issues arising from supporting information (other than missing files) should be addressed to the authors.

Supporting Information

## Data Availability

The data that support the findings of this study are available in the supplementary material of this article.
