# Supplementary material for: Experiment and Theory Clarify: Sc+ Receives One Oxygen Atom from SO2 to Form ScO+, which Proves to be a Catalyst for the Hidden Oxygen‐Exchange with SO2
Source: Chemphyschem. 2022 Jan 20;23(5):e202100773. doi: 10.1002/cphc.202100773 (PMC9303259; doi:10.1002/cphc.202100773)
Supplement: Supplementary file 1 — Supporting Information [file CPHC-23-0-s001.pdf]

ChemPhysChem

Supporting Information

Experiment and Theory Clarify: Sc^+ Receives One Oxygen Atom from SO_2 to Form ScO^+, which Proves to be a Catalyst for the Hidden Oxygen-Exchange with SO_2

Jose M. Mercero,* Elixabete Rezabal, Jesus M. Ugalde, Thomas Weiske, and Jilai Li*

Supporting Information

Contents

1 Experimental Details **1**

2 Computational Details **2**

3 Tables **4**

4 References **8**

1 Experimental Details

The ion/molecule reactions were performed in a Spectrospin CMS 47X Fourier transform ion cyclotron resonance (FT-ICR) mass spectrometer equipped with an external ion source as described elsewhere.[1] Briefly, Sc^+ was generated by laser ablation of a scandium target using a Nd:YAG laser operating at 532 nm; helium served as a cooling and carrier gas. It is important that the helium pipe has been baked beforehand to remove impurities on the inner walls of the feeding pipes and thus significantly improve the production of Sc^+. Using a series of ion lenses, the ions were transferred into the ICR cell, which is positioned in the

bore of a 7.05 T superconducting magnet. After thermalization by about 1×10^5 collisions with pulsed-in argon, the reactions of mass-selected Sc^+ were studied by introducing sulfur dioxide (SO_2) via leak valves at stationary pressures. In the $[Sc^{18}O]^+/SO_2$ experiments, trace amounts of $^{18}O_2$ were mixed to the helium used for cooling down the scandium plasma by supersonic expansion of the noble gas inside the external ion source for the generation of $[Sc^{18}O]^+$. A temperature of 298 K was assumed for the thermalized clusters.[1]

The rate constants have been determined following the detailed protocol documented in the PhD Thesis of K. Koszinowski.[3] Typically, the pressures are determined with an uncalibrated Bayard-Alpard ion gauge whose reading differs depending on the kind of the gas. As the concentration of the ionic reactant A^+ is small compared to the neutral substrate B, a pseudo first-order reaction can be assumed as a good approximation,

$$\frac{d[A^+]}{dt} = -k[A^+][B] \cong -k_{obs}[A^+], \text{ with} - k_{obs} = -k[B] \quad (1)$$

here, k is the true bimolecular and k_{obs} represents the apparent pseudo-unimolecular rate constant. Recording a time-dependent profile of the natural logarithm of the normalized intensity of the educt ions delivers the decline of the reactant ions whose negative slope corresponds to k_{obs}. For a general procedure to determine reaction-rate constants of ions with neutrals in the diluted gas phase, see reference.[4]

2 Computational Details

The basis functions set used for scandium was the TZVP basis set of Alrichs et al [5] supplemented with a diffuse s function, two sets of pure p functions (optimized by Watchers [6]), one set of diffuse pure d angular momentum functions (optimized by Hay [7]), and three sets of uncontracted pure angular momentum f functions including both tight and diffuse exponents, as recommended by Raghavachari and Trucks [8]. For the oxygen and sulfur atoms, the aug-cc-pVTZ basis set was used [9,10]. This basis set has been previously reported to provide a satisfactory balance between reliability and computational feasibility for similar scandium cation reactivity studies [11]. For convenience, this basis set will be named TZVP+ throughout this paper.

Calculations for the $Sc^+ + SO_2 \rightarrow [ScO]^+ + SO$ oxygen abstraction reaction have been carried out at the high-level wavefunction-based complete active space self-consistent field method, supplemented with Multiconfigurational Quasi Degenerate Perturbation Theory calculations in order to improve the energetics.

We have optimized the geometries (and diagonalized the Hessian at the optimized geometries in order to check for the correct number of negative eigenvalues, i.e.: zero for the minima and one for the transition states) of the stationary points for both the singlet and triplet potential energy surfaces at the multiconfigurational self-consistent field [12] (MCSCF) level of theory with the TZVP+ basis set. The active space contained fourteen active electrons in fifteen orbitals, which include the 2p orbitals of the oxygens, the 3p's of sulfur, and the 3d and 4s orbitals of scandium. It will be hereafter denoted as MCSCF(14,15)/TZVP+. The optimized geometrical data are shown in Table S1.

Subsequently, to account for the dynamic electron correlation missed in the MCSCF procedure, multiconfigurational quasi degenerate perturbation theory [13] (MCQDPT) calculations were carried out on the MCSCF(14,15)/TZVP+ optimized geometries. All valence and virtual orbitals have been correlated in the MCQDPT calculations. Inconsistencies caused by the so-called intruder state, which appear when the perturbation expansion of the reference MCSCF wavefunction has vanishingly small energy denominators, were remedied by shifting them by 0.02 a.u., as recommended earlier [14]. The resulting relative energies are shown in Table S2.

Spin-orbit coupling effects have been explicitly considered for the accurate estimation of the singlet-triplet relative energy between the ^{3}D and ^{1}D terms of Sc^+ arising from the lowest energy electronic configuration $3p^6 3d^1 4s^1$. The spin-orbit coupling splits the ^{3}D line into three states denoted by their corresponding total angular momentum quantum number J=1,2,3. Table S3 shows that our theoretical estimates lie satisfactorily close to their corresponding experimental marks.

Addtionally, nonadiabactic spin-orbit couplings between the singlet and triplet potential energy surfaces, considered in this research, have been studied by means of the spin-orbit coupling constants as estimated by the Pauli-Breit hamiltonian [15,16], including both the one- and two-electron contributions. The spin-orbit coupling calculations have been carried out with non-orthogonal molecular orbitals with the same core orbitals for both states and optimized active space molecular orbitals. The initial states are pure spin functions, and off-diagonal elements have been introduced through the Pauli-Breit hamiltonian. Consequently, the reported spin-orbit coupling constants correspond to the diagonalized elements of the spin-orbit hamiltonian. However, notice that the initial states are used to designate coupling constants. The characterization of the geometry of the minimum energy crossing points (MECP) has been carried out on the same line at which the singlet and triplet adiabatic surfaces intersect. The optimized geometries of the two MECP's are shown in Table S4, along with the estimated spin-orbit coupling constants. All calculations alluded to above have been carried out with the GAMESS program [17].

Calculations for the $[Sc^{18}O]^+ + SO_2 \rightarrow [ScO]^+ + SO^{18}O$ oxygen-exchange reaction have been carried out at the quantum chemistry gold-standard method for single-configuration systems, i.e.: the CCSD(T) method [18]. Optimization of the geometries and subsequent inspection of the number of negative eigenvalues of the diagonalized Hessian, was carried out at the B3LYP level of theory [19]. We selected the TZVP+ basis functions set based on the excellent performance of this CCSD(T)/TZVP+//B3LYP/TZVP+ level of theory for the reactivity of first-row transition metals with water [20].

Table S5 shows the calculated CCSD(T)/TZVP+//B3LYP/TZVP+ T1-diagnostics values [21] for all the five stationary geometries characterized on the reaction path of the $[Sc^{18}O]^+ + SO_2 \rightarrow [ScO]^+ + SO^{18}O$ reaction. A recent thoughtful study of first-row transition metal containing compounds suggests that species with T1-diagnostics < 0.05, met the requirements for reliable single reference CCSD(T) calculations [22]. This requirement is amply met by all our structures, consequently, single reference wavefunction-based electronic structure theory, CCSD(T), stands as the method of choice for the accurate mechanistic description of the $[Sc^{18}O]^+ + SO_2 \rightarrow [ScO]^+ + SO^{18}O$ reaction. The optimized geometries are shown in Table S6.

All CCSD(T) and B3LYP calculations have been carried out with the Gaussion16 program [23].

3 Tables

Table S1. Optimized geometries of the stationary points of the reaction path for the Sc^+ + SO_2 → $[ScO]^+$ + SO oxygen abstraction reaction at the MCSCF(14,15)/TZVP+ level of theory, in Å, for both the singlet and triplet potential energy surfaces.

Encounter Complex, EC_R1. Singlet

O	0.000000	0.425015	0.000000
S	-1.573871	-0.057702	0.000000
O	-1.566305	-1.510799	0.000000
Sc	1.795827	0.457595	0.000000

Encounter Complex, EC_R1. Triplet

O	0.000000	0.425015	0.000000
S	-1.573871	-0.057702	0.000000
O	-1.566305	-1.510799	0.000000
Sc	1.795827	0.457595	0.000000

Transition State TS1. Singlet

O	0.000000	0.647470	0.000000
S	-1.552962	0.129326	0.000000
O	-1.471447	-1.327208	0.000000
Sc	1.743761	0.160414	0.000000

Transition State TS1. Triplet

O	-0.074437	-0.767334	0.000000
S	1.480475	-0.360649	0.000000
O	1.514772	1.098780	0.000000
Sc	-1.676680	0.148515	0.000000

Four Center Complex, IM1. Singlet

O	0.507761	0.0084673	-1.2275765
S	0.449815	-1.0940123	0.0000000
O	0.507761	0.0084673	1.2275765
Sc	-0.759044	0.8313987	0.0000000

Four Center Complex, IM1. Triplet

O	0.000000	1.2084248	-0.3752305
S	0.000000	0.0000000	-1.3589569
O	0.000000	-1.2084248	-0.3752305
Sc	0.000000	0.0000000	1.3599179

```
Transition State TS2. Singlet

 O    -0.931321     2.765452     0.000000
Sc     0.004122     0.914096     0.000000
 S    -0.298114     4.099651     0.000000
 O     1.534152     1.417928     0.000000

Transition State TS2. Triplet

 O     0.513482    -1.092368     0.000000
Sc    -1.248743    -0.120967     0.000000
 S     1.429808     0.097883     0.000000
 O    -0.095148     1.214141     0.000000

Exit Channel Complex, EC_P1. Singlet

 O    -0.636908     3.002245     0.000000
 S    -0.848300     4.491823     0.000000
Sc     0.075977     0.861418     0.000000
 O     1.718076     0.841636     0.000000

Exit Channel Complex, EC_P1. Triplet

 O    -0.756530     2.972821     0.000000
 S    -0.758794     4.473834     0.000000
Sc     0.088756     0.889180     0.000000
 O     1.735412     0.861291     0.000000
```

Table S2. Relative energies of the stationary structures of the $Sc^+ + SO_2 \rightarrow [ScO]^+ +$ SO reaction at the MCQDPT/TZVP+//MCSCF(14,15)/TZVP+ level of theory, in kJ/mol. The reference energy has been set at $Sc^+(^3D_1, 3p^63d^14s^1) + SO_2(^1A_1)$, -1307.741071000 a.u.

	Triplet	Singlet
Sc^++SO_2	0.00	28.65
$\mathbf{EC}_{R1}$	-131.80	-129.34
$\mathbf{TS}_1$	-102.13	-94.06
$\mathbf{IM}_1$	-203.81	-258.53
$\mathbf{TS}_2$	-36.13	-86.02
$\mathbf{EC}_{P1}$	-261.86	-99.33
ScO^++SO	-120.04	-42.24

Table S3. Experimental and calculated, MCQDPT/TZVP+, relative energies, in cm^{-1}, of the spin-orbit coupled energy level arising from the $3p^63d^14s^1$ electronic configuration of Sc^+.

			Experimental[a,b]	Calculated
Configuration	Term	J	Level (cm^{-1})	Level (cm^{-1})
$3p^63d^14s^1$	^3D	1	0.00	0.00
		2	67.72	67.42
		3	177.76	176.15
$3p^63^1d4s^1$	^1D	2	2540.95	2434.54

[a] J. Sugar, C. Corliss, J. Phys. Chem. Chem. Ref. Data, **1985**, *14*, Suppl. 2, 1–664

[b] A. Kramida, Yu. Ralchenko, J. Reader, and NIST ASD Team (2020). NIST Atomic Spectra Database (version 5.8), [Online]. Available: https://physics.nist.gov/asd [Wed Oct 20 2021]. National Institute of Standards and Technology, Gaithersburg, MD. DOI: https://doi.org/10.18434/T4W30F

Table S4. Optimized geometries, in Å, and spin-orbit coupling parameters $\langle H_{SO} \rangle$, in cm^{-1}, of **MECP0**, **MECP1** and **MECP2** minimum energy crossing points between the singlet and triplet potential energy surfaces of the $Sc^+ + SO_2 \rightarrow [ScO]^+ + SO$ reaction.

MECP0, $\langle H_{SO} \rangle$=0.94 cm^{-1}

O	0.000000	-1.178545	-0.375063
S	-0.000000	-0.002796	-1.350305
O	0.000000	1.186803	-0.380823
Sc	-0.000000	-0.005461	1.356690

MECP1, $\langle H_{SO} \rangle$=0.03 cm^{-1}

O	-0.160943	0.884503	0.000000
S	-1.583873	-0.079020	0.000000
O	-1.220137	-1.542168	0.000000
Sc	1.620603	0.050794	0.000000

MECP2, $\langle H_{SO} \rangle$=234.07 cm^{-1}

O	1.693465	1.153566	0.000000
S	0.814561	3.567567	0.000000
Sc	0.053084	0.777285	0.000000
O	-0.535640	2.815780	0.000000

Table S5. CCSD(T)/TZVP+ energies, in a.u., and T1-diagnostics values of the B3LYP/TZVP+ optimized structures for the $[Sc^{18}O]^+ + SO_2 \rightarrow [ScO]^+ + SO^{18}O$ reaction on the singlet potential energy surface. $\mathbf{TS}_3$ stands for the Transition State of main text's Fig. 3

	Energy	T1-diagnostics
$[Sc^{18}O]^+$ ($^1\Sigma^+$)	-834.7574746	0.03690493
SO_2 ($^1A_1$)	-547.9908513	0.02187440
$\mathbf{EC}_{R2}([Sc^{18}O]^+ \cdots SO_2)$	-1382.7975007	0.02742056
$\mathbf{TS}_3$	-1382.7957218	0.02737043
$\mathbf{IM}_2([OS(\eta^2O_2Sc]^+)$	-1382.8022716	0.02794865

Table S6. Optimized geometries, in Å, of the stationary points of the reaction path for the $[Sc^{18}O]^+ + SO_2 \rightarrow [ScO]^+ + SO^{18}O$ reaction at the B3LYP/TZVP+ level of theory.

```
Encounter Complex, EC_R2.

 O                   2.089442    1.093180   -0.439317
Sc                   1.683419   -0.331910    0.242460
 O                  -0.446393   -0.610948   -0.149941
 S                  -1.858170   -0.182158   -0.339630
 O                  -2.345686    0.753348    0.632058

Transition State, TS3.

 O                  -1.299811    1.298515   -0.203557
Sc                  -1.621224   -0.274214    0.155669
 O                   0.378783   -0.984553   -0.112931
 S                   1.461695    0.013103   -0.409460
 O                   2.253351    0.379645    0.726778

Four Center Complex, IM2.

Sc                   0.813786   -1.217495    0.000000
 S                  -0.862491    0.800268   -0.000000
 O                  -0.137069    2.039497   -0.000000
 O                  -0.137069   -0.222053    1.210793
 O                  -0.137069   -0.222053   -1.210793
```

4 References

[1] (a) M. Engeser, T. Weiske, D. Schröder, H. Schwarz, J. Phys. Chem. A 2003, 107, 2855-2859; (b) D. Schröder, H. Schwarz, D. E. Clemmer, Y. Chen, P. B. Armentrout, V. I. Baranov, D. K. Böhme, Int. J. Mass Spectrom. 1997, 161, 175-191; (c) K. Eller, H. Schwarz, Int. J. Mass Spectrom. 1989, 93, 243-257.

[2] (a) C. Wang, X.-K. Gu, H. Yan, Y. Lin, J. Li, D. Liu, W.-X. Li, J. Lu, ACS Catal. 2017, 7, 887-891; (b) Z. Wu, G. Hu, D.-e. Jiang, D. R. Mullins, Q.-F. Zhang, L. F. Allard, L.-S. Wang, S. H. Overbury, Nano Lett. 2016, 16, 6560-6567; (c) C. Doornkamp, V. Ponec, J. Mol. Catal. A: Chem. 2000, 162, 19-32; (d) P. Mars, D. W. van Krevelen, Chem. Eng. Sci. 1954, 3, 41-59; (e) C. Geng, J. Li, T. Weiske, H. Schwarz, J. Am. Chem. Soc. 2018, 140, 9275-9281; (f) C. Geng, J. Li, T. Weiske, H. Schwarz, Proc. Natl. Acad. Sci. USA 2019, 116, 21416-21420.

[3] K. Koszinowski, PhD thesis, Technische Universität Berlin (Berlin), 2003.

[4] A. G. Marshall, S. E. Buttrill, J. Chem. Phys. 1970, 52, 2752-2759.

[5] A. Schafer, C. Huber, R. Alrichs, J. Chem. Phys., 1992, 97, 2571-2577: ibid, 1994, 100, 5829-5835.

[6] A. J. H. Watchers, J. Chem. Phys. 1970, 52, 1033-1036.

[7] P. J. Hay, J. Chem. Phys. 1977, 66, 4377-4384.

[8] K. Raghavachari, G. W. Trucks, J. Chem. Phys. 1989, 91, 1062-1065.

[9] R. A. Kendakk, T. H. Dunning, R. J. Harrison, J. Chem. Phys. 1992, 96, 6796-6806.

[10] D. E. Woon, T. H. Dunning, J. Chem. Phys. 1993, 98, 1359-1371.

[11] A. Irigoras, J. E. Fowler, J. M. Ugalde, J. Am. Chem. Soc. 1999, 121, 574-580

[12] A. C. Wahl, G. Das. "The Multiconfigurational Self-Consistent Method", in "Methods of Electronic Structure Theory" (Ed.: H. F. Schaefer III) Plenum Press, New York, 1997

[13] H. Nakano, J. Chem. Phys. 1993, 99, 7983-7992.

[14] H. A. Witck, Y. K. Choe, J. P. Finley, K. Hirao, J. Comp. Chem. 2002, 23, 957-966.

[15] H. A. Bethe, E. E. Salpeter, "Quantum Mechanics of one- and two-electron Atoms", Springer-Verlag, Berlin, 1957, pp. 170-205.

[16] D. G. Fedorov, M. S: Gordon, J. Chem. Phys. 2000, 112, 5611-5623

[17] M. W. Schmidt, K. K. Baldrige, J. A. Boatz, S. T. Elbert, M. S. Gordon, J. H. Jensen, S. Koseki, N. Matsunaga, K. A. Nguyen, S. J. Su, T. L. Windus, M. Dupuis, J. A. Montgomery, J. Compt. Chem. 1993, 14, 1347-1363.

[18] D. J. Cramer, "Essentials of Computational Chemistry", Chischester: John Wiley &

Sons, Ltd., 2002, pp. 191-232.

[19] A. D. Becke, J. Chem. Phys. 1993, 98, 5648-5652.

[20] A. Irigoras "Water Dehydrogenation by First-Row Tansition Metals Cations: A Paradigm for Two-State Reactivity", Ph. D. Thesis. 1999. ISBN: 84-8373-197-5. University of the Basque Country Press. http://www.ehu.eus/chemistry/theory/Files/tesi_arantxa.pdf

[21] T. J. Lee, P. R. Taylor, Int. J. Quantum Chem., Quant. Chem. Symp. 1989, S23, 199-207.

[22] W. Jian, N. J. DeYonker, J. J. Determan, A. K. Wilson, J. Chem. Phys. A, 2002, 116. 870-885.

[23] Gaussian 16, Revision C.01, M. J. Frisch, G. W. Trucks, H. B. Schlegel, G. E. Scuseria, M. A. Robb, J. R. Cheeseman, G. Scalmani, V. Barone, G. A. Petersson, H. Nakatsuji, X. Li, M. Caricato, A. V. Marenich, J. Bloino, B. G. Janesko, R. Gomperts, B. Mennucci, H. P. Hratchian, J. V. Ortiz, A. F. Izmaylov, J. L. Sonnenberg, D. Williams-Young, F. Ding, F. Lipparini, F. Egidi, J. Goings, B. Peng, A. Petrone, T. Henderson, D. Ranasinghe, V. G. Zakrzewski, J. Gao, N. Rega, G. Zheng, W. Liang, M. Hada, M. Ehara, K. Toyota, R. Fukuda, J. Hasegawa, M. Ishida, T. Nakajima, Y. Honda, O. Kitao, H. Nakai, T. Vreven, K. Throssell, J. A. Montgomery, Jr., J. E. Peralta, F. Ogliaro, M. J. Bearpark, J. J. Heyd, E. N. Brothers, K. N. Kudin, V. N. Staroverov, T. A. Keith, R. Kobayashi, J. Normand, K. Raghavachari, A. P. Rendell, J. C. Burant, S. S. Iyengar, J. Tomasi, M. Cossi, J. M. Millam, M. Klene, C. Adamo, R. Cammi, J. W. Ochterski, R. L. Martin, K. Morokuma, O. Farkas, J. B. Foresman, and D. J. Fox, Gaussian, Inc., Wallingford CT, 2016.
